# Fishing for happiness: The effects of generating positive imagery on mood and behaviour

**DOI:** 10.1016/j.brat.2011.10.003

**Published:** 2011-12

**Authors:** Arnaud Pictet, Anna E. Coughtrey, Andrew Mathews, Emily A. Holmes

**Affiliations:** a Department of Psychiatry, University of Oxford, UK; b Department of Psychology, University of California, Davis, CA, USA

**Keywords:** Mental imagery, Emotion, Depression, Behaviour, Evaluative learning

## Abstract

Experimental evidence using picture–word cues has shown that generating mental imagery has a causal impact on emotion, at least for images prompted by negative or benign stimuli. It remains unclear whether this finding extends to overtly positive stimuli and whether generating positive imagery can increase positive affect in people with dysphoria. Dysphoric participants were assigned to one of three conditions, and given instructions to generate mental images in response to picture–word cues which were either positive, negative or mixed (control) in valence. Results showed that the positive picture–word condition increased positive affect more than the control and negative conditions. Participants in the positive condition also demonstrated enhanced performance on a behavioural task compared to the two other conditions. Compared to participants in the negative condition, participants in the positive condition provided more positive responses on a homophone task administered after 24 h to assess the durability of effects. These findings suggest that a positive picture–word task used to evoke mental imagery leads to improvements in positive mood, with transfer to later performance. Understanding the mechanisms underlying mood change in dysphoria may hold implications for both theory and treatment development.

## Highlights

► A mental imagery generation task was tested on a sample of dysphoric participants. ► We examined changes in depressed mood, behaviour and cognitive bias. ► Positive, negative and mixed valence (control) imagery conditions were contrasted. ► Positive imagery led to an improvement in mood and behaviour.

Can we engage imagination to promote more positive mood? Recent evidence using picture–word cues suggests that evoking mental imagery has a more powerful impact on emotion than using verbal language (Holmes, Mathews, Mackintosh, & Dalgleish, 2008), at least for negative and benign stimuli. This is consistent with a line of research suggesting that mental imagery has a special impact on emotion (Arntz, de Groot, & Kindt, 2005; Holmes & Mathews, 2005, 2010; Holmes, Mathews, Dalgleish, & Mackintosh, 2006). However, the impact of generating mental imagery from overtly positive picture–word stimuli remains to be explored. We know little about the promotion of overtly positive imagery, particularly when this is most needed as in depressed mood. While the research is part of a broader endeavour to improve depressed mood, in this study we focused on the extent to which positive affect (rather than depressed mood per se) improved as a result of generating positive imagery. Our focus on positive affect and imagery was driven by recent findings that depression is not just associated with an excess of negative cognitions and affect, but also a deficit in positive cognitions (Holmes, Lang, Moulds, & Steele, 2008; Joorman, Siemer, & Gotlib, 2007; MacLeod & Byrne, 1996), positive affect (Watson, Clark, & Carey, 1988) and willingness to pursue potentially rewarding goals (Dickson, Moberly, & Kinderman, 2011).

Research suggests that individuals with depression fail to benefit from the emotional lift provided by evoking past and future positive personal events (Joorman et al., 2007; Joormann & Siemer, 2004). Several mechanisms may contribute to this effect. With respect to mental imagery, this lack of congruent emotional response might be explained by three characteristic features of depressive thinking. First, depression has been shown to be associated with a deficit in generating positive imagery about the future (Holmes, Lang, et al., 2008; Stöber, 2000; Williams et al., 1996). Second, depression is characterized by rumination which is predominantly verbal in nature (Fresco, Frankel, Mennin, Turk, & Heimberg, 2002). The verbal processing of positive information can have the paradoxical effect of worsening mood rather than improving it (Holmes, Lang, & Shah, 2009). Finally, depressed individuals tend to recall positive autobiographical events from an observer rather than a field perspective (Lemogne et al., 2006). Imagining positive scenarios from an observer perspective has been shown to lower mood, possibly due to unfavourable comparisons with perceived reality (Holmes, Coughtrey, & Connor, 2008). For all these reasons, we have suggested the utility of promoting positive future-oriented, field perspective mental imagery for improving depressed mood (Blackwell & Holmes, 2010; Holmes, Lang, & Shah, 2009).

The benefit of generating images about positive future outcomes may not be limited to mood and cognitive bias improvement but extend to subsequent behaviour. Indeed, a body of evidence suggests that imagining one’s own future behaviour can increase the likelihood of that behaviour being enacted in reality (Carroll, 1978; Gregory, Cialdini, & Carpenter, 1982; Pham & Taylor, 1999). Imagining a possible event outcome has been shown to increase subjective probability that the same outcome will actually occur (Carroll, 1978). Depressed individuals’ attitudes towards the future is marked with reduced expectancies for positive events (MacLeod, Rose, & Williams, 1993; Pysczcynski & Greenberg, 1987) and a lack of approach motivation towards future goals (Dickson & MacLeod, 2004). Moreover, a clinical feature of depression is a lack of interest in almost all everyday activities that were previously perceived as rewarding and enjoyable (American Psychiatric Association, 2000). Consequently, individuals with depression are less persistent and tend to engage less time in goal-directed behaviours than do healthy individuals (Hopko, Armento, Cantu, Chambers, & Lejuez, 2003; Hopko & Mullane, 2008).

Previous studies of dysphoric and depressed individuals have shown that, although these groups are as able as non-dysphoric groups to identify desirable activities and goals, they are more pessimistic about the outcome of any attempts to achieve these goals (Dickson et al., 2011). Similarly, when actually required to attempt experimental tasks, dysphoric individuals (and those exposed to learned helplessness inductions) are more readily discouraged by anticipated loss, are more inclined to give up trying after failures, and are less encouraged by expected rewards, resulting in poorer performance when effort is required to achieve success (Henriques, Glowacki, & Davidson, 1994; Miller & Seligman, 1975; Pizzagalli, Iosifescu, Hallett, Ratner, & Fava, 2008). On the basis of these results, we predicted that the performance of dysphoric participants would be improved by positive imagery designed to increase the anticipation of success under ambiguous conditions, thus enhancing their effortful engagement in a difficult but potentially rewarding task. A fishing game task (as in Reinecke, Deeprose, & Holmes, submitted for publication) was chosen as a simple behavioural performance measure assumed to tap behaviour negatively associated with dysphoria, such as approach motivation and persistence, while being clearly different in nature from the picture–word task.

Exploring the transfer of positive mental imagery to behaviour is of both clinical and theoretical relevance (Kavanagh, Andrade, & May, 2005). The deficit in motivated behaviour is one of the most pernicious symptoms in depression because it cuts the individual off from pleasant and rewarding experiences. This behavioural component of depression has long been targeted in Cognitive Behavioural Therapy (CBT) and has also given rise to the development of successful interventions known as “behavioural activation” (Jacobson, Martell, & Dimidjian, 2001). The aim of behavioural activation is to increase the likelihood of engaging in rewarding behaviours. Therefore, the clinical relevance of using an experimental task designed to encourage positive mental imagery to alleviate depressed mood would be further supported if a behaviour-enhancing effect followed training.

In the current experiment, dysphoric participants were given training and instructions to evoke mental imagery in line with our previous work on mental imagery generation (Holmes, Mathews, et al., 2008). Participants were then asked to imagine and rate the vividness of imagery in response to viewing 200 picture–word combinations within one of three conditions: positive resolution, negative resolution or a mixed control condition, before completing the behavioural task. To assess the durability of effects, a measure based on the perceived meaning of emotionally ambiguous homophones (Mathews, Richards, & Eysenck, 1989) was completed 24 h later.

Our predictions were that, compared to the control condition, the positive condition would lead to a greater increase in positive mood. Conversely, we predicted that compared to control condition, the negative condition would lead to a greater decrease in positive mood. Furthermore, we predicted that generating mental images in response to picture–word cues would impact on later behaviour, with participants in the positive condition performing better on the fishing task than those in the control and negative conditions. Finally, we predicted a similar pattern of results on the homophone test completed 24 h later.

## Method

### Participants

Eighty-seven individuals (50 women; mean age = 27.96, SD = 11.33) with scores in the dysphoric range on the Beck Depression Inventory (BDI-II; Beck, Steer, & Brown, 1996) were recruited via advertisements in the local University town. When defining dysphoric samples it is generally recommended to use a multistage process (Kendall, Hollon, Beck, Hammen, & Ingram, 1987; Wenzlaff & Bates, 1998) to ensure that negative mood is not merely elevated at the start of the experimental session. In this study we defined dysphoric mood as a BDI-II (BDI-II; Beck, Steer, & Brown, 1996) score of 10 or above, at two time points, separated by at least 5 days, following Aarts, Wegner, and Dijksterhuis (2006), Dalgleish and Yiend (2006) and Sedek and von Hecker (2004). Thus, of the 87 participants meeting criteria at recruitment, 81 still met criteria at the beginning of the experimental session and were included in the study. The 6 people that no longer had a BDI-II score of 10 or above at the beginning of the experimental session (*M* = 8.00, SD = 1.55) did not differ from the sample in terms of age, *t*(85) = 1.06, *p* = .29, or gender *χ*^2^ (2, *N* = 87) = 3.57, *p* = .60. The mean BDI-II score for the final sample of 81 participants was 17.63 (SD = 7.56) at recruitment, and 16.52 (SD = 7.21) at the beginning of the experimental session (henceforth referred to as time 1 in Table 1).

### Materials

#### Picture–word cues to generate mental images

Participants were required to generate mental images in response to picture–word cues as in earlier research (Holmes, Mathews, et al., 2008), for more details see the Procedure. Presentation of the picture–word cue stimuli was controlled using E-Prime software, version 1.1.4.1 (Schneider, Eschman, & Zucclotto, 2002) running on a Dell PC (P791). The set of 200 digital pictures used were photographed for the study by local art students given the remit of producing ambiguous pictures, or by the authors using a Sony digital camera, or downloaded from the internet (non-copyrighted). The pictures were 640 × 480 pixels saved as Bitmap files. They were displayed, centred on the 17″ VDU such that their height took up 85% of the VDU height.

Each picture was of common everyday objects and scenes from the local area that a student might typically encounter (e.g. students attending a lecture, partying with friends, sporting activities). Each picture was paired with a word or short phrase intended, when paired with the relevant picture, to create a positive emotional resolution in the positive condition (e.g. a picture of students attending a lecture, and the word “understanding”) or negative emotional resolution in the negative condition (e.g. the same picture with the word “struggling”). In the control condition, half of the pictures were paired with captions from the negative condition, and the remaining 50% were captions from the positive condition (similar to the approach of Hirsch, Hayes, & Mathews, 2009). All participants saw the same pictures, but the captions accompanying them differed accordingly to their assigned condition. During the imagery generation phase, each word was displayed in white against a black background, centrally beneath the relevant picture, using type font Arial at point size 30, for 3500 ms. During the liking ratings (see below), the pictures were displayed without words against a black background.

#### Filler task to equalize mood

An unrelated task of listening to a series of 40 s classical music extracts was administered during a 10-min interval after the imagery generation phase. This task has been successfully used in previous studies (Holmes, Lang, & Shah, 2009; Holmes et al., 2006) to minimize the influence of mood differences across groups and return mood to baseline before post-training assessment tasks. Participants rated how pleasant they found each musical extract on a scale of 1 (*extremely unpleasant*) to 9 (*extremely pleasant*).

#### Homophone task

A list of 14 homophones (e.g. dye/die) and 14 unambiguous neutral words of similar word frequency was used (based on Mathews et al., 1989; Mogg, Bradbury, & Bradley, 2006). The task was chosen for reasons of convenience since it could be delivered by telephone 24 h after the experimental session. The 24 h gap between the experimental manipulation and the interpretation bias measure was selected in line with previous investigation of the durability of interpretation bias modification procedure (Yiend, Mackintosh, & Mathews, 2005). The experimenter read the homophones over the phone one word at a time. Participants were required to report the spelling of each word in turn. The total number of negative spellings (e.g. die rather than dye) was calculated and converted into a percentage of the number of all correctly spelled homophones.

#### Questionnaire measures

The trait tendency to use mental imagery was measured using the 12-item Spontaneous Use of Imagery Scale (SUIS: Reisberg, Pearson, & Kosslyn, 2003). Each item was rated on a 5-point scale (never appropriate to always completely appropriate).

Depressive symptoms were measured using the 21-item Beck Depression Inventory-II (BDI-II; Beck, Steer, & Brown, 1996) in which participants responded to a series of questions on a scale from 0 to 3. Possible scores range from 0 to 63, with 0–13 classified as minimal depression and 14–19 as mild depression. The BDI-II possesses high internal consistency with an alpha level of .9 (Beck, Steer, Ball, & Ranieri, 1996). One week test retest reliability is also high, *r* = .93 (Beck, Steer, & Brown, 1996). In addition, hopelessness was measured using the 20-item Beck Hopelessness Scale (BHS; Beck & Steer, 1998) rated on a dichotomous scale (*true*–*false*).

Positive affective state was measured using the combined positive affect subscales (21-items) of the Positive and Negative Affect Schedule (PANAS; Watson, Clark, & Tellengen, 1988) as in previous work (e.g. Holmes, Mathews, et al., 2008). Each word was rated on a 5-point scale (*very slightly or not at all* to *extremely*), using the short-term time instructions (“Indicate to what extent you feel this way now/in the past few minutes”).

#### Behavioural task: the fishing game

This simple task employed a toy measuring 1500 mm × 900 mm × 250 mm, in which 12 brightly coloured plastic fish moved round in a circle, opening and closing their mouths to reveal a magnet. Participants were required to catch as many fish as they could in 2.5 min by “hooking” them using a magnet on the end of a 900 mm plastic fishing rod. The authors used 2 different sets of fishing games of identical make and model.

#### Valence ratings

Valence ratings for 50 pictures (in the absence of words) were obtained before and after the imagery generation task (as in Holmes, Mathews, et al., 2008). To sample across the 200 trials, the 50 pictures consisted of a sub-selection of approximately 10 taken from each of the 5 picture–word training blocks, presented in a randomized order. Participants rated the emotional valence of each picture from 1(*extremely unpleasant*) to 9 (*extremely pleasant*) using a standard keyboard.

#### Subjective experience questions

As a further manipulation check, participants were asked a series of questions about their subjective experience of performing the picture–word task, both after the first and last blocks of training. Questions assessed task difficulty (“How difficult or easy did you find your task of combining the picture and the word”), use of verbal processing and imagery during the training phase (“How much were you verbally analyzing/imagining the picture–word combinations?”) and use of field perspective (“How much were you imagining the picture–word trough your own eyes”). Questions were rated on 9-point scales, ranging from 1 (not at all) to 9 (extremely/all the time).

### Procedure

Participants gave their written informed consent and were randomly assigned to positive, negative or control conditions (*N* = 27 per condition). They completed the BDI-II (for the second time to confirm dysphoric status), trait STAI, SUIS, BHS and PANAS (time 1). Then participants completed the valence ratings of 50 pictures presented on the computer.

The mental imagery generation instructions, similar to those used in Holmes, Mathews, et al. (2008) were then read out: In all conditions, mental imagery was described as “the experience of seeing with the mind’s eye, hearing with the mind’s ear, and so on…”, using Kosslyn, Ganis, and Thompson’s (2001) definition i.e. in any sensory modality. All participants were given an imagery generation practice task of imagining cutting a lemon. The difference between field (first person) and observer (third person) imagery was explained using two photographs of a person in the laboratory taken from either a field or observer perspective (as in Holmes, Coughtrey, et al., 2008). Participants were asked to imagine the combination of each picture and word from a field perspective (i.e. seeing the situation through their own eyes, as if they were actively involved). The experimenter also explained how it was possible to imagine anything, even if it did not immediately seem believable, using a picture of a flying elephant as an example.

In the next stage, practice was given in generating mental imagery in response to picture–word cues. Two picture–word combinations (one positive and one negative) were given on paper as practice examples by the experimenter. Next, four picture–word items, consistent with condition assignment were presented on the computer as full practice examples (as in Holmes, Mathews, et al., 2008). Participants were told that they were to produce a field perspective mental image combining the picture with the word. Instructions on the computer screen for 1000 ms stated “imagine the combination of the next picture and word as if you were actively involved”. The picture and word then appeared for 3500 ms, followed by a 1000 ms beep. Participants were asked to shut their eyes and imagine, and to describe their practice images aloud while the experimenter checked that the description of the mental image included reference to both picture and word. To promote compliance with the imagery task instructions, after each picture–word trial, participants had 10 s in which they made a rating of mental image vividness on a scale from 1 (*not at all vivid*) to 9 (*extremely vivid*). Participants then continued to the remaining practice items.

#### Main experimental task

Participants were asked to generate mental images (as defined and practised above) in response to each of a series of picture–word cues. Two hundred picture–word cues were presented in 5 randomized blocks of 40 according to condition. As in the practice trials, instructions, presented on the computer screen for 1000 ms, stated “imagine the combination of the next picture and word as if you were actively involved.” The picture–word cue was displayed for 3500 ms, during which participants were asked to shut their eyes and imagine the stimulus as soon as they saw it. This was followed by a 1000 ms beep, at which point they had to open their eyes and make a rating of image vividness on a scale from 1 (*not at all vivid*) to 5 (*extremely vivid*) to promote task compliance in generating imagery. Participants were prompted to make their ratings as quickly as possible without thinking too much about their answers. Reminder instructions were given in between blocks.

The PANAS was re-administered at the end of the imagery generation phase (time 2). Following this there was a 10 min filler task before the PANAS was re-administered (time 3). Participants then completed valence ratings for 50 pictures each displayed alone for 1500 ms, in a separate random order for each participant, followed by the behavioural task in which they were given the fishing game and asked to catch as many fish as they could in 2.5 min. Finally participants completed the manipulation check ratings and left the laboratory. At 24 h after the experimental session, the experimenter telephoned each participant and administered the homophone task. Finally, all participants were debriefed and thanked.

## Results

An alpha level of .05 was used for all statistical tests.

### Randomization checks

Comparisons of participant characteristics assigned to each condition revealed no significant differences in terms of gender (11 men in the positive condition, 9 men in the negative condition, and 11 men in the benign condition); age, *F*(2, 78) = .72, *p* = .49; BDI-II at recruitment, *F*(2, 78) = .16, *p* = .86; BDI-II at baseline Time 1, *F*(2, 78) = .19, *p* = .98; BHS, *F*(2, 78) = .27, *p* = .77; SUIS, *F*(2, 78) = .66, *p* = .52; STAI-trait, *F*(2, 78) = .48, *p* = .62 (see Table 1); Time 1 PANAS, *F*(2, 78) = .10, *p* = .91; or baseline liking ratings for the pictures, *F*(2, 78) = 2.32, *p* = .11. Time 1 PANAS correlated significantly in our sample with BDI-II at baseline, *r*(81) = −.53, *p* < .001.

### Positive affect change over the training phase

PANAS scores were analyzed in a mixed model ANOVA with a within-subjects factor of time (pre- vs. post-training) and a between-subjects factor of condition (positive vs. negative vs. control). As predicted, there was a significant interaction of time with condition, *F*(2, 78) = 14.31, *p* < .001, *η*^2^ = .27. There was no main effect of time *F*(2, 78) = .20, *p* = .66, *η*^2^ = .03 or condition *F*(2, 78) = 1.93, *p* = .15, *η*^2^ = .05.

**Fig. 1:**
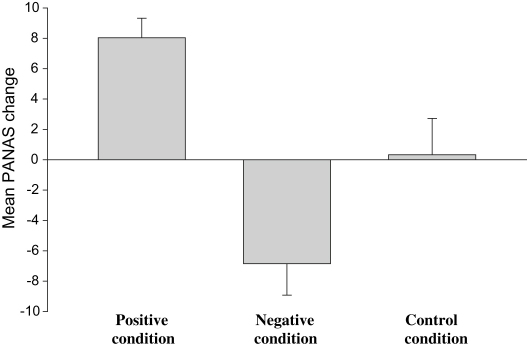
Mean changes in positive affect (PANAS) from pre to post-training in the each condition. Error bars show one standard error of the mean.

To facilitate interpretation, we computed change scores for each condition, and decomposed the interaction between time and condition using independent samples *t*-tests, comparing change scores for each training group with the control condition. As predicted, there was a greater increase in positive affect for the positive than the control condition (*M* = 8.04, SD = 6.65 vs. *M* = .33, SD = 12.42), *t*(52) = 2.84, *p* = .006, *d* = .77. Conversely, there was a greater *decrease* in positive affect for the negative than the control condition (*M* = −6.85, SD = 10.74 vs. *M* = .33, SD = 12.42), *t*(52) = 2.27, *p* = .027, *d* = .62 (Fig. 1 illustrates the PANAS changes within each condition).

Paired sample *t*-tests were used to test whether within each group there had been the predicted change in affect from pre to post-training (following Holmes et al., 2006). As predicted, the positive condition showed a significant increase in positive affect, *t*(26) = 6.28, *p* < .001, *d* = .50, whereas the negative group showed a significant decrease in positive affect *t*(26) = 3.32, *p* = .003, *d* = .51. There were no significant changes in PANAS scores across training within the neutral condition, *t*(26) = .14, *p* = .89.

### Vividness ratings during the training phase

Vividness rating scores were analyzed in a mixed model ANOVA with a within-subjects factor of time (pre- vs. post-training) and a between-subjects factor of condition (positive vs. negative vs. control). There was no main effect of time *F*(2, 78) = 2.83, *p* = .096, *η*^2^ = .03 or condition *F*(2, 78) = .77, *p* = .47, *η*^2^ = .02, but there was a significant interaction of time with condition, *F*(2, 78) = 3.45, *p* = .037, *η*^2^ = .08. This interaction was decomposed by comparing the vividness means over time separately for each group using paired sample *t*-tests. Participants in the positive condition showed significant increases in vividness ratings *t*(26) = 2.87, *p* = .008, *d* = .35 but there were no significant changes in vividness ratings in either the negative, *t*(26) = .60, *p* = .55, *d* = .06 or the control condition *t*(26) = .45, *p* = .66, *d* = .06.

### Affect after the filler task

For the PANAS administrated after the filler task, a one-way ANOVA revealed no significant difference between conditions *F*(2, 78) = .12, *p* = .89, thus indicating equivalence prior to administration of the behavioural task.

### Behavioural task

As described above, there were two different sets of the fishing game, with equal numbers of participants in each condition using the two sets (15 participants in each condition used the first set and 12 used the second set). Unfortunately, there were unanticipated differences between scores achieved with the two sets (*M* = 39.19, SD = 4.06 vs. *M* = 21.62, SD = 5.29), *t*(79) = 16.44, *p* < .001, *d* = 3.73, presumably due to physical differences between the two sets, such as a stronger fishing rod magnet in one set. After running an ANOVA to check that game set did not interact with training condition *F*(2) = 1.91, *p* = .16, *η*^2^ = .05, the game set was entered as a covariate in an ANCOVA.

The total number of fish caught between the three conditions was compared using an ANCOVA with the game set entered as a covariate. There was a significant effect of training condition, *F*(2, 77) = 12.85, *p* < .001, *η*^2^ = .25. Independent *t*-tests were used to run planned comparisons between the individual conditions, calculated using the estimated means and degrees of freedom adjusted appropriately for an ANCOVA (Tabachnick & Fidell, 1996, p. 347). As predicted, participants in the positive training condition caught significantly more fish than participants in the control condition (*M*_adj_ = 32.41, SD = 4.19 vs. *M*_adj_ = 29.26, SD = 4.19), *t*(77) = 2.76, *p* = .007, *d* = .75. Significantly more fish were caught in the positive than in the negative condition (*M* = 26.63, SD = 4.19), *t*(77) = 5.06, *p* < .001, *d* = 1.38. In line with predictions, participants in the negative condition caught significantly less fish than those in the control condition, *t*(77) = 2.30, *p* = .02, *d* = .63.

### Homophone task

A one-way ANOVA revealed a significant difference in the percentage of negative spellings made between groups, *F*(2, 78) = 5.21, *p* = .008, *η*^2^ = .12. Independent sample *t-*tests did not show the predicted difference between positive and control conditions *t*(52) = 1.34, *p* = .19, *d* = .36, although participants in the negative condition were significantly more negative than those in the control condition (*M* = 68.79, SD = 12.24 vs. *M* = 62.70, SD = 9.37), *t*(52) = 2.05, *p* = .045, *d* = .56. Furthermore, as predicted, participants in the positive condition perceived fewer negative meanings than participants in the negative condition (*M* = 58.47, SD = 13.48 vs. *M* = 68.79, SD = 12.24), *t*(52) = 2.95, *p* = .005, *d* = .80.

### Valence ratings

Valence ratings of the pictures before and after training were analyzed in a mixed model ANOVA that revealed a significant interaction of time with condition *F*(2, 78) = 9.69, *p* < .001, *η*^2^ = .20. There was no main effect of time, *F*(2, 78) = .02, *p* = .89, *η*^2^ = .00, but a main effect of condition, *F*(2, 78) = 8.42, *p* < .001, *η*^2^ = .18. Change scores were computed for each condition and the interaction between time and condition was decomposed using independent samples *t*-tests. In line with expectations, there was a greater increase in valence ratings in the positive compared to the control condition (*M* = .43, SD = .73 vs. *M* = −.07, SD = .51), *t*(52) = 2.89, *p* = .006, *d* = .79, and compared to the negative condition (*M* = −.37, SD = .80), *t*(52) = 3.83, *p* < .001, *d* = 1.04. Any difference between the negative and the control conditions did not reach significance, *t*(52) = 1.68, *p* = .10, *d* = .45.

Paired sample *t*-tests were used to test whether within each group there had been the predicted change in valence ratings from pre to post-training. As predicted, the positive condition showed a significant increase in valence ratings *t*(26) = 3.03, *p* = .005, *d* = .65, whereas the negative group showed a significant decrease in valence ratings *t*(26) = 2.56, *p* = .02, *d* = .62. There were no significant changes in valence ratings across training reported within the control condition, *t*(26) = .69, *p* = .50, *d* = .09.

### Subjective experience questions

Subjective experience of the training was recorded at the end of the first and last training block. Responses were compared between conditions using one-way ANOVAs. There was no difference between groups in ratings for use of imagery, either at the end of the first block of training, (*M* = 7.78, SD = 1.05 in the positive condition: *M* = 7.63, SD = 1.50 in the negative condition; *M* = 7.48, SD = 1.44 in the control condition), *F*(2, 78) = .32, *p* = .73, or at the end of the last block of training, (*M* = 7.67, SD = 1.04 in the positive condition: *M* = 7.48, SD = 1.34 in the negative condition; *M* = 7.66, SD = 1.44 in the control condition), *F*(2, 78) = .19, *p* = .83. The use of field imagery, *M* = 7.63, SD = 1.40, *F*(2, 78) = .06, *p* = .95, ease of the task, *M* = 5.84, SD = 1.73, *F*(2, 78) = .67, *p* = .52 and low use of verbal processing, *M* = 3.33, SD = 1.92, *F*(2, 78) = .13, *p* = .88 was also comparable at the end of the last training block.

## Discussion

The present results suggest that systematic practice in generating overtly positive mental imagery from picture–word cues can help increase positive affect in people with dysphoria. This finding is relevant to the suggestion that a deficit in positive future-oriented imagery in depression may impede recovery from depressed mood (Holmes, Lang, & Deeprose, 2009; Williams, Healy, & Ellis, 1999). Mood improved in the positive condition, deteriorated in the negative condition and remained stable in the control condition. These differences cannot be attributed to pre-training mood differences, as mood scores were equivalent between groups at baseline. Further, the impact of the imagery conditions was corroborated on a task in which participants rated valence for associated pictures presented without captions, perhaps due to effects akin to evaluative conditioning (cf. Holmes, Mathews, et al., 2008; Standage, Ashwin, & Fox, 2009).

In addition, participants in the positive imagery condition performed better on a behavioural task (a fishing game) than did those in the two other conditions. Differences in performance were unlikely to be due to variations in state mood, since this was successfully equalized after the 10-min filler task. Finally, as predicted there were indications that the procedure influenced subsequent emotional interpretation. The pictures alone can be considered a form of ambiguous stimuli, in that the word captions steer their interpretation as positive or negative. Forming mental images in response to positive picture–word cues led to congruent effects on interpretations of homophones made after a 24 h time delay, with participants in the positive condition perceiving fewer negative meanings than those in the negative condition. However, it should be noted that the difference between positive and control conditions did not reach significance.

As highlighted by MacLeod, Koster, and Fox (2009) in a recent commentary on cognitive bias modification research (CBM), the consequences of most CBM studies have been assessed via self report measures of state and trait mood, which may be prone to experimenter demand effects and contamination via priming of congruent mood descriptors. The present study to some extent addresses this methodological limitation. First, affect change following our procedure was accompanied by parallel changes on an indirect measure used as a manipulation check (e.g. valence ratings for associated stimuli). Second, we showed transfer of effects to performance on a behavioural task completed within a controlled experimental setting. Here again, demand effects remain possible but seem unlikely given the lack of any obvious similarity between mental imagery generation and the fishing game task. Finally, there was evidence of some transfer of training to a completely new task (i.e. interpretation of homophones) that was quite different from the format of the experimental procedure.

A concern that might be raised about these results is that the average scores on the fishing game were unexpectedly found to differ depending on which set was used, probably due to one set having a more powerful magnet that the other. However, equal numbers of participants in each condition were assigned to each set, and this factor did not interact significantly with condition, so it seems that set assignment did not influence the main finding of differences between conditions.

The current study has a number of limitations. One important limitation is that participants were only mildly depressed and (beyond the limited data on the homophone task) only relatively immediate effects of imagery were assessed. Thus, we cannot be certain that more severely depressed individuals would respond positively, nor how long any effects would persist. Also, in the absence of any comparison between imagery and alternative forms of positive representation we cannot be certain that the present method is the best method of achieving mood and performance improvements; for example, other tasks involving “deep” levels of processing might have similar effects. Although this work follows on other studies using the same imagery generation task in comparison to a verbal procedure (e.g. Holmes, Mathews, et al., 2008), the absence of such a comparison with a non-imagery condition in the current study does not allow us to conclude that mental imagery per se was responsible for the obtained effects. However, several prior comparisons of imagery with verbal representations have consistently found imagery to have stronger effects on emotional outcomes, with verbal methods sometimes having paradoxical negative effects. Further, our data on manipulation check questions suggest that our participants engaged in imagery to a similar extent, irrespective of the condition they were allocated to. For those in the positive condition, images became more vivid as they went along with the training, which supports our assumption that the imagery task improved participant’s ability to generate positive images.

Another limitation of the present study was while changes in state positive affect were examined as a result of undertaking the experimental task, negative affect was not assessed. Consistent with previous findings (Watson et al., 1988), positive affect scores in our sample were significantly correlated with baseline levels of depression. However, given that positive and negative affect are commonly regarded as two separable and independent aspects of emotional experience, future studies should include a state measure of negative as well as positive affect to encompass both components of depressive experience.

A further limitation is that we used a measure of lasting effects (the homophone task) that is potentially susceptible to response bias effects (Mogg et al., 2006). This task was selected in part because it could be delivered over the telephone, but other measures such as the Scrambled Sentence Test (Rude, Valdez, Odom, & Ebrahimi, 2003) might have provided a convergent test of interpretation bias. Also, we did not test whether participants’ ability to generate positive imagery improved as a result of the training. Future research could benefit from investigating this issue using measures such as the Prospective Imagery Task (Stöber, 2000). It is possible that our findings of enhanced behavioural engagement and reduced negative interpretations of homophones following our procedure would have been strengthened by including a baseline assessment of these variables. However, in addition to the classical reasons for relying on random allocation to an experimental administration rather than employing change scores (see Cronbach & Furby, 1970) our use of post-manipulation assessment was motivated by the likelihood that both critical outcome measures would be influenced by practice or the recall of previous responses. A challenge for future research is to develop experimental tasks that would be more suitable for use in repeated measure designs (e.g. Berna, Lang, Goodwin, & Holmes, 2011).

Despite these reservations, the finding of enhanced performance in the positive mental imagery group may hold clinical relevance. One of the core symptoms of depression is anhedonia (American Psychiatric Association, 2000), which refers to both a diminished ability to experience pleasure and reduced motivation to seek reward. Our findings suggest that practice in generating positive mental imagery may be used not only to increase emotional responding to positive and rewarding stimuli, but could also increase the likelihood of achieving these goals. Future research would profitably be extended to test transfer to a greater range of behavioural goals and tasks, and further examine the underlying mechanism(s) by which generating positive imagery improve later behavioural performance.

Only one possible method of promoting positive mental imagery was studied here, and more needs to be known about the comparative effectiveness of alternative methods. This is particularly true in the case of individuals with depression, who can fail to benefit from the emotional lift provided by evoking positive personal events (Joorman et al., 2007; Joormann & Siemer, 2004). Our findings are limited to showing that people with dysphoric mood benefit in both mood and behaviour from a task involving practice in generating positive imagery. Consequently, it will be important to investigate the extent to which the present conclusion can similarly be applied to depressive disorders. The effects of generating positive mental imagery on increasing positive affect in dysphoria may have potentially useful clinical implications and the current study represents an initial step towards that end.

## Figures and Tables

**Table 1:** Characteristics of participants at baseline per condition.

Characteristic	Positive (*n* = 27)		Negative (*n* = 27)		Control (*n* = 27)	
	*M*	SD	*M*	SD	*M*	SD
Age (years)	29.78	12.37	28	12	26	9.50

Gender (%)						
Female	59		67		59	
Male	41		33		41	

BDI-II						
At recruitment	18.15	7.96	17.00	6.98	17.74	7.94
At baseline (time 1)	16.67	6.85	16.30	7.14	16.59	7.87

BHS	6.04	4.59	6.00	5.43	6.89	5.10
STAI-T	46.33	11.23	46.41	11.93	48.96	10.38
SUIS	41.15	6.52	38.63	8.39	39.85	9.10

*Note*: BDI-II = Beck Depression Inventory-II, BHS = Beck Hopelessness, STAI = Spielberger State-Trait Anxiety Inventory, SUIS = Spontaneous Use of Imagery Scale, PANAS = total positive affect score from the PANAS.
